# Outcome and quality of life after aorto-bifemoral bypass surgery

**DOI:** 10.1186/1471-2261-10-15

**Published:** 2010-03-18

**Authors:** Fernando J Abelha, Miguela Botelho, Vera Fernandes, Henrique Barros

**Affiliations:** 1 Department of Anesthesia, Hospital de São João, Porto, Portugal; 2 Department of Hygiene and Epidemiology - University of Porto Medical School Porto - Portugal

## Abstract

**Background:**

Aorto-bifemoral bypass (AFB) is commonly performed to treat aorto-iliac disease and a durable long-term outcome is achieved. Most studies documenting beneficial outcomes after AFB have been limited to mortality and morbidity rates, costs and length of hospital stay (LOS). Few studies have examined the dependency of patients and how their perception of their own health changes after surgery. The aim of the present study was to evaluate outcome after AFB and to study its determinants.

**Methods:**

This retrospective study was carried out in the multidisciplinary Post-Anaesthesia Care Unit (PACU) with five intensive care beds. Out of 1597 intensive care patients admitted to the PACU, 75 were submitted to infrarenal AFB and admitted to these intensive care unit (ICU) beds over 2 years. Preoperative characteristics and outcome were evaluated by comparing occlusive disease with aneurysmatic disease patients. Six months after discharge, the patients were contacted to complete a Short Form-36 questionnaire (SF-36) and to have their dependency in Activities of Daily Living (ADL) evaluated. Patient's characteristics and postoperative follow-up data were compared using Mann-Whitney U test, t test for independent groups, chi-square or Fisher's exact test. Patient preoperative characteristics were evaluated for associations with mortality using a multiple logistic regression analysis.

**Results:**

The mortality rate was 12% at six months. Multivariate analysis identified congestive heart disease and APACHE II as independent determinants for mortality. Patients submitted to AFB for occlusive disease had worse SF-36 scores in role physical and general health perception. Patients submitted to AFB had worse SF-36 scores for all domains than a comparable urban population and had similar scores to other PACU patients. Sixty-six percent and 23% of patients were dependent in at least one activity in instrumental and personal ADL, respectively, but 64% reported having better general health.

**Conclusion:**

This study shows that congestive heart disease and APACHE II were risk factors for mortality after AFB surgery. Survivors who have undergone AFB perceive an improved quality of life although they are more dependent in ADL tasks and have worse scores in almost all SF-36 than the population to which they belong.

## Background

Although some studies have documented beneficial outcome after aorto-bifemoral bypass (AFB) surgery, most have been limited to mortality and morbidity rates, cost and length of hospital stay (LOS) [1,2]. Few studies have examined the dependency of these patients and how they perceive changes in their own health after this procedure, and little is known about the extent and impact of these changes on patient outcome.

In this study we review the characteristics of patients undergoing AFB, studying outcomes up to six months after the procedure, when dependency on Activities of Daily Living (ADL) and health related quality of life were evaluated. For this study we considered patients who were submitted to scheduled or emergent surgery for either an abdominal aortic aneurysm or arterial occlusive disease.

We considered the stratification of patients according to cardiac risk factors [3] which some authors regard as predictors of mortality or LOS.

Several questionnaires have been validated for the study of Health Related Quality of Life [4-8]. Most of the measures that have been used are multi-item scales; that is, they comprise several questions or items. Some multiple-item scales provide a total score as well as generating subscales that provide information on particular aspects such as mobility. The Short-Form General Health Survey (SF-36) was developed during the Medical Outcomes Study (MOS) to measure general health concepts relevant across age, disease and treatment groups [9]. It is a self-completed questionnaire covering all aspects of Health Related Quality of Life (HRQOL). It is a valid instrument for measuring HRQOL. It has been used for post-discharge ICU patients and for studying groups with other diseases; it shows good reliability and validity [9,10]. This questionnaire was culturally adapted to Portuguese and validated in a study by Ferreira[11,12].

Low functional status puts patients at higher risk. Patients with only minor or no clinical predictors but with poor functional capacity are recommended to undergo noninvasive testing prior to this surgery. The ability to care for oneself and live independently has been considered a measure of functional outcome after hospitalization [13]. Functional status refers to the level of involvement in activities and is often used as a synonym for performance in ADL [14]. ADL appraisal scales consider functional and instrumental activities. A patient's ability to handle these activities has been assessed by generic or disease-specific measures of physical functional status. Katz's Activities of Daily Living Scale[14] and the Lawton Instrumental Activities of Daily Living [15] have been investigated in critical care survivors.

The determination of functional outcome and the identification of predictors of survival and functional recovery after AFB may be fundamental for evaluating the needs of these patients and promoting proper treatment.

The aim of the present study was to evaluate quality of life and independence in activities of daily living in patients submitted to AFB surgery.

## Methods

The Institutional Review Board of the Hospital de São João approved the study protocol and written consent was obtained from the patients or members of their families.

This retrospective cohort study was carried out in the multidisciplinary Post-Anaesthesia Care Unit (PACU) at the Hospital São João, an 1124-bed community teaching hospital in Porto, Portugal, over a period of two years beginning in March 2006. Included in the PACU was a Surgical Intensive Care Unit with five beds to which critically ill surgical patients are admitted and are closely monitored and treated.

All consecutive postoperative patients admitted to the surgical ICU area of the PACU, who were submitted electively for aorto-bifemoral bypass surgery for either infrarenal abdominal aortic aneurysm or arterial occlusive disease, were enrolled. Patients who were readmitted during the study period were included only on the first admission.

Patients were excluded from the study if the procedure was performed to treat a symptomatic or ruptured abdominal aortic aneurysm or if cross-clamping above the renal arteries was required.

The following clinical variables were recorded on admission to the ICU: age, sex, body weight and height and ASA physical status. At admission, the core temperature was registered using a tympanic thermometer and a sample of blood was taken to measure troponin I. The ICU and in-hospital LOS and mortality were also recorded for all patients, and the Score of Simplified Acute Physiology Score (SAPS) II [16] and Acute Physiology & Chronic Health Evaluation (APACHE) II [17] were calculated using standard methods.

Specifically, preadmission comorbidities, any history of ischaemic heart disease, congestive heart disease, cerebrovascular disease, hypertension, renal insufficiency, diabetes or hyperlipidaemia were recorded. The presence of coexisting conditions was assessed using the secondary diagnoses of the International Classification of Diseases, Ninth Revision, Clinical Modification (ICD-9-CM). Adapting a classification scheme developed by Lee and colleagues [3], we calculated a Revised Cardiac Risk Index (RCRI) score for each patient, assigning one point for each of the following risk factors (defined as in the original description made by Lee et al.): high-risk surgery, ischaemic heart disease, congestive heart disease, cerebrovascular disease, renal insufficiency and diabetes mellitus.

### Functional capacity

Functional capacity before surgery was evaluated by the ability to handle personal and instrumental ADL, assessed by a questionnaire that evaluates the functional independence of the individual in performing personal activities of daily living (P-ADL) and instrumental activities of daily living (I-ADL). This evaluation was completed again at six months after PACU discharge; on the same occasion, the patients completed a questionnaire on Health-Related Quality of Life.

### Medical Outcome Survey Short-Form 36 (SF-36)

Health-related quality of life was assessed by the Medical Outcomes Study Short-form Health Survey (SF-36) [10]. This contains 36 questions that evaluate eight health domains considered to be important for patient well-being and health status. These domains reflect physical health, mental health, and the impact of health on daily functioning. The eight multiple-item domains encompass physical functioning (ten items), social functioning (two items), role limitations caused by physical problems (four items), role limitations caused by emotional problems (three items), mental health (five items), energy and vitality (four items), pain (two items) and general perception of health (five items). There is one further unscaled item that addresses self-reported changes in the respondent's health status during the past year. For each item, scores are coded, summed and transformed to a scale from 0 (worst possible health state measured by the questionnaire) to 100 (best possible health state). Scores can be aggregated to measures representing a physical health summary scale (consisting of physical functioning, physical role, pain and general health) and a mental health summary scale (vitality, social functioning, emotional role and mental health) [4].

The answers to the question in SF-36 about self-reported changes in health status ("compared to one year ago, how you would rate your health in general now?") were dichotomized as better, about the same or worse than one year ago.

To minimize distress to the next of kin, each patient's records were checked on the hospital information system after six months to ascertain whether he or she was still alive. A copy of a formal letter was sent to all known survivors accompanied by a return envelope and a validated Portuguese SF-36 self-report form [11,12]. This version of the SF-36 has been validated for the study population in the Porto region from which the subjects of this report were drawn [18]. Scores for all domains obtained for patients after AFB were compared with the published [18] values for this urban population.

We also compared the scores on all SF-36 domains for patients after AFB surgery with scores obtained for other PACU patients who were not submitted to AFB surgery.

### Activities of Daily Living (ADL)

The personal ADL considered were bathing, dressing, going to the toilet, transferring from bed to chair, continence and feeding. The instrumental ADL considered were cleaning, food shopping, public transportation and cooking. This questionnaire was based on Katz' Index of Independence in ADL [19] and Lawton IADL [15] scales. Answers were also categorized into two groups, able or unable to perform each activity or group of activities. Patients were classified by their ability to perform physical and psychosocial ADL and four categories were possible: (a) I-ADL and P-ADL independent, (b) I-ADL dependent but P-ADL independent, (c) P-ADL dependent but I-ADL independent and (d) both P-ADL and I-ADL dependent.

### Outcome

The outcome endpoints considered were: (1) Functional capacity and ADL - patients were considered dependent whether they were dependent in at least one I-ADL or P-ADL activity; (2) Quality of life - quality of life was evaluated at six months after PACU discharge; (3) Mortality - patients were considered survivors if they were alive six months after PACU discharge.

### Statistical methods

Descriptive analyses of variables were used to summarize data and the Mann-Whitney U test was used to compare continuous variables between two groups of subjects; chi-square or Fisher's exact test was used to compare proportions between two groups of subjects.

To identify independent predictors of mortality univariate analysis was performed using simple binary logistic regression with an odds ratio (OR) and 95% confidence interval (CI) with the following independent variables: type of surgery, age, gender, BMI, ASA-PS, troponin I blood levels and temperature at admission to the PACU, comorbidities, RCRI score, duration of anaesthesia, and severity of disease scores (APACHE II and SAPS II). All variables were deemed to be significant if *P *< 0.05.

Multiple regression binary logistic with forward conditional elimination was used to examine covariates and to identify independent predictors of mortality. In this model covariates with a univariate *p *≤ 0.05 in the univariate analysis were entered.

The Mann-Whitney U test and "t test" for independent groups were used to compare population means.

SPSS for Windows version 16.0 (SPSS, Chicago, IL) was used to analyze the data.

## Results

During the study period there were 1597 admissions in the PACU and 75 patients met the inclusion criteria. The characteristics of all patients enrolled in the study are given in Table 1.

**Table 1:** Patient characteristics and outcome

Variable	AFB (n = 75)	ANE (n = 27)	AOD (n = 48)	P
**Age in years, median (IQR)**	**64 (57-72)**	**70 (64-75)**	**59(53-68)**	**<0.001^c^**
**Age group, n (%)**				**0.001^a^**
**≥ **65 years	36 (48)	20 (74)	16 (33)	
< 65 years	39 (52)	7 (26)	32 (67)	
Sex, n (%)				0.406^b^
Male	73 (97)	27(100)	46 (96)	
Female	2 (3)	0 (0)	2 (4)	
ASA physical status				0.174^b^
I/II	26 (35)	7 (26)	19 (40)	
III/IV	49 (65)	20 (74)	29 (60)	
**Body Mass Index in Kg/m**^2^**, median (IQR)**	**24 (21-27)**	**26 (24-31)**	**23 (21-26)**	**0.004^c^**
Duration of anaesthesia (min.), median (IQR)	300 (270-360)	300 (270-360)	300(264-357)	0.519^c^
**Temperature at PACU admission, median (IQR)**	**34.5 (33.4-35.9)**	**34.2 (32.5-35.1)**	**34.8 (33.9-36.0)**	**0.029^c^**
**Troponin I at PACU admission, median (IQR)**	**0.09 (0.01-0.04)**	**0.04 (0.01-0.16)**	**0.01 (0.01-0.04)**	**0.029^c^**
Hypertension, n (%)	55 (73)	23 (85)	32 (67)	0.068^b^
Hyperlipidemia, n (%)	37 (49)	16 (59)	21 (44)	0.197^a^
High-risk surgery, n (%)	75 (100)	27 (100)	48 (100)	-
Ischaemic heart disease, n (%)	32 (43)	11 (41)	21 (44)	0.800^a^
Congestive heart disease, n (%)	19 (25)	6 (22)	13 (27)	0.431^b^
Cerebrovascular disease, n (%)	7 (9)	1 (4)	6 (13)	0.204^b^
Renal insufficiency	3 (4)	1(4)	2 (4)	0.707^b^
Insulin therapy for diabetes, n (%)	3 (4)	1(4)	2 (4)	0.707^b^
Total RCRI				0.440^a^
≤ 2	35(47)	11 (41)	24 (50)	
Katz scale, median (IQR)	0 (0-0)	0 (0-0)	0 (0-0)	0.459^c^
Dependency in I-ADL, n (%)	1 (1)	0 (0)	1 (2)	0.646^b^
Lawton I-ADL, median (IQR)	7 (7-7)	7 (7-7)	7 (7-7)	0.241^c^
Dependency in P-ADL, n (%)	6 (8)	1 (4)	5 (10)	0.290^b^
**SAPS II, median (P25-75)**	**18 (13-27)**	**23 (18-33)**	**15 (12-22)**	**0.002^c^**
**APACHE II, median (IQR)**	**9 (7-13)**	**11 (8-15)**	**9 (6-11)**	**0.012^c^**
PACU length of stay (hours), median (IQR)	44 (36-68)	43 (21-102)	44 (40-68)	0.603^c^
Hospital length of stay (days), median (IQR)	11 (8-28)	11 (8-24)	11 (8-30)	0.475^c^
Mortality in PACU, n (%)	3 (4)	0 (0)	3 (6)	0.256^b^
Mortality in hospital, n (%)	7 (9)	2 (7)	5 (10)	0.507^b^
Mortality at 6 months follow-up	9 (12)	4 (14)	5 (10)	0.414^b^

^a ^Pearson χ^2^, ^b ^Fisher's exact test, ^c^Mann-Whitney U test, IQR, interquartile range

AFB, aorto-bifemoral bypass surgery; ANE, aneurysmatic disease; AOD, aortic occlusive disease

RCRI, Revised Cardiac Risk Index I-ADL, Instrumental Activities of Daily Living; P-ADL, Personal Activities of Daily Living; SAPS II, Simplified Acute Physiology Score, APACHE II Acute Physiology & Chronic Health Evaluation PACU, Post Anaesthesia Care Unit; IQR, interquartile range

Ninety-seven percent were male. The median age was 64 years (minimum 41, maximum 80), median SAPS II was 18 (range 7-69) median APACHE II was 9 (range 4-25) and median LOS in the PACU was 44 hours. Seven patients (9%) died during hospital stay.

Forty-eight patients had complex aorto-iliac occlusive disease and surgery was performed on 27 patients to treat aortic aneurysm.

Compared to those who had ABF surgery because of occlusive aortic disease, patients submitted to ABF surgery for correction of abdominal aortic aneurysm were older (median 70 versus 59 years, *P *< 0.001), had higher BMI (median 26 versus 23, *P *= 0.004), had lower temperature (median 34.2 versus 34.8, P = 0.029) and higher troponin I at admission to the PACU (median 0.04 versus 0.01, P = 0.029) and were more severely ill (median SAPS II 23 versus 15, *P *= 0.002 and median APACHE II 11 versus 9, *P *= 0.012).

Sixty-eight patients were discharged from hospital; two died before the six-month evaluation (12% global mortality at the time of evaluation). Of the remaining 66 patients, 16 (24%) did not answer the questionnaires at six months follow-up but were known to be alive.

The characteristics of all patients who were still alive at six-month follow-up are presented in Table 2. Of these 50 participants, 98% were male and the median age was 64 years, median SAPS II was 18, median APACHE II was 9 and median PACU LOS was 43 hours. There were no statistically significant differences between participants and non-participating patients in respect of the variables studied.

**Table 2:** Comparison of characteristics between respondents and non-respondents

Variable	Respondents (n = 50)	Non-respondents (n = 16)	P value
Type of surgery			0.407^b^
AFB for Aneurysmatic disease	19 (37)	4 (29)	
AFB for occlusive disease	31 (63)	12 (71)	
Age in years, median (IQR)	64 (56 - 70)	61 (55 - 70)	0.884^c^
Sex, n (%)			0.452^b^
Male	48 (98)	16 (94)	
Female	1 (2)	1 (6)	
BMI (Kg/m^2^), median (IQR)	23 (20 - 24)	25 (22 - 27)	0.393^c^
Duration of anaesthesia (min.), median (IQR)	300 (270 - 336)	300 (240 - 360)	0.729^c^
Temperature at admission, median (IQR)	34.5 (33.2 - 35.6)	34.5 (33.3 - 36.0)	0.084^c^
ASA Physical status			0.481^a^
I/II	18 (37)	7 (41)	
III/IV	31 (63)	10 (59)	
Troponin at admission, median (IQR)	0.04 (0.01 - 0.04)	0.01 (0.01 - 0.05)	0.780^b^
Total RCRI			0.442^a^
≤ 2	26 (53)	8 (47)	
>2	23 (47)	9 (53)	
Hypertension n.(%)	36 (74)	13 (77)	0.541^b^
Hyperlipidemia, n (%)	22 (45)	11 (65)	0.130^b^
Ischaemic heart disease	23 (47)	6 (35)	0.293^b^
History of congestive heart disease	11 (22)	2 (12)	0.283^b^
History of cerebrovascular disease	1 (2)	2 (11)	0.050^b^
Insulin therapy for diabetes	3 (6)	0(0)	0.403^b^
Renal insufficiency	1 (2)	0 (0)	0.742^b^
Dependency in P-ADL	1 (2)	0 (0)	0.817^b^
Dependency in I-ADL	6 (12)	0 (0)	0.279^b^
Previous Katz, median (IQR)	0 (0 - 0)	0 (0 - 0)	0.636^c^
Previous Lawton, median (IQR)	7 (7 - 7)	7 (7 - 7)	0.226^c^
SAPS II, median (IQR)	18 (13 - 24)	15 (12 - 23)	0.643^c^
APACHE II, median (IQR)	9 (7 - 13)	8 (5 - 11)	0.121^b^
Hours of ICU length of stay, median (IQR)	43 (23 - 68)	44 (40 - 56)	0.467^c^
Days of hospital length of stay, median (IQR)	11 (8 - 20)	15 (8 - 32)	0.210^c^

^a ^Pearson χ^2^, ^b ^Fisher's exact test, ^c^Mann-Whitney U test, IQR, interquartile range

AFB, aorto-bifemoral bypass surgery; BMI, Body Mass Index; RCRI, Revised Cardiac Risk Index; I-ADL, Instrumental Activities of Daily Living; P-ADL, Personal Activities of Daily Living; SAPS II, Simplified Acute Physiology Score; APACHE II Acute Physiology & Chronic Health Evaluation PACU; PACU, Post Anaesthesia Care Unit.

### Mortality

Patients submitted to ABF because of aortic occlusive disease had higher mortality rates at the PACU (6% versus 0%) and during hospital stay (10% versus 7%), but had lower mortality rates (10% versus 14%) at six months follow-up. However, these differences were not statistically significant.

Univariate analysis identified the following independent predictors for mortality at 6 months follow-up (Table 3): age (OR 1.15, 95% CI 1.03 to 1.28, *P *= 0.015), congestive heart disease (OR 8.15, 95% CI 1.80 to 37.01, *P *= 0.007), renal insufficiency (OR 18.57, 95% CI 1.49 to 231.7, *P *= 0.023) and RCRI score (OR 8.50, 95% CI 1.01 to 71.83, *P *= 0.049 for RCRI > 2), SAPS II (OR 1.09, 95% CI 1.04-1.14, p = 0.001) and APACHE II (OR 1.51, 95% CI 1.17-1.83, p = 0.001).

**Table 3:** Univariate analysis for determinants of mortality after aorto-bifemoral bypasses surgery

	nonsurvivors/survivors		
Variable	n = 9 n = 66	Odds ratio (95% CI)	P
Type of AFB surgery			
for Aneurysmatic disease	4 (44)/23 (35)	1.50 (0.36 - 6.12)	0.575
for occlusive disease	5 (56)/43 (65)	1	
**Age**	**73 (67-76)/55 (62-70)**	**1.15 (1.03 - 1.28)**	**0.015**
Gender		-	0.999
Female	0 (0)/2 (3)		
Male	9 (100)/64 (97)		
BMI, median	20 (21-29)/24 (21-26)	0.96 (0.74 - 1.24)	0.733
Duration of anaesthesia (min.)	333(330-450)/300 (264-357)	1.01 (1.00 - 1.01)	0.117
ASA Physical status			
I/II	1 (11)/25(38)	1	
III/IV	8 (89)/41(62)	4.88 (0.58 - 41.36)	0.146
Temperature at admission	34.3(34.0-35.1)/34.5(33.3-35.9)	0.96 (0.61 - 1.50)	0.848
Troponin at admission	0.01(0.01-0.05)/0.02(0.01-0.04)	0.001 (0 - 3500)	0,656
Hypertension	6(67)/49 (74)	0.63 (0.16 - 3.09)	0.631
Hyperlipidemia	4 (44)/33(50)	0.80 (0.20 - 3.25)	0.755
Ischaemic heart disease	3 (33)/29(44)	0.64 (0.15 - 2.77)	0.549
**Congestive heart disease**	**6 (67)/13 (20)**	**8.15 (1.80 - 37.01)**	**0.007**
Cerebrovascular disease	2 (22)/5(8)	3.49 (0.57 - 21.45)	0.178
Insulin therapy for diabetes	0 (0)/3 (5)	-	0.999
**Renal insufficiency**	**2 (22)/1 (2)**	**18.57 (1.49 - 231.7)**	**0.023**
**RCRI**			
≤ 2	1(11)/34 (52)	1	
>2	8 (89)/32 (49)	8.50 (1.01 - 71.83)	0.049
Previous score in Katz scale	0/0	-	0.999
Previous score in Lawton scale	7 (7-7)/7(4-7)	0.76 (0.48-1.18)	0.215
**SAPS II, median**	**33 (19-60)/18 (13-23)**	**1.09 (1.04 - 1.14)**	**0.001**
**APACHE II**	**13 (15-25)/8 (6-11)**	**1.51 (1.17 - 1.83)**	**0.001**
Length of PACU stay (hours)	93 (45-140)/44 (34-66)	1.01 (1.00 - 1.02)	0.066
Length of Hospital stay (days)	9 (8-32)/11(8-27)	1.00 (0.98 - 1.02)	0.990

AFB, aorto-bifemoral bypass surgery; BMI, Body mass index; ASA, American Society of anesthesiologists, COPD, Chronic obstructive pulmonary disease; RCRI, Revised cardiac risk index; PACU, Post Anaesthesia Care Unit, SAPS, Simplified Acute Physiology Score; APACHE II Acute Physiology & Chronic Health Evaluation

Multiple logistic regression analysis with forward conditional elimination was used to examine covariate effects of each factor on mortality (Table 4). The regression model included all variables with statistical significance in the univariate analysis for determinants of mortality. This analysis showed that independent risk factors for hospital mortality, after adjustment for age, congestive heart disease, renal insufficiency, RCRI, SAPS II and APACHE II were congestive heart disease (OR 9.86, 95% CI 1.48 to 65.55, *P *= 0.018), and APACHE II (OR 1.40, 95% CI 1.11 to 1.76, *P *= 0.005).

**Table 4:** Multivariate regression analysis for predictors of mortality.

Variable	Simple OR	p	Adjusted* OR (95% CI)	p*
Age	1.15	0.015		
Renal insufficiency	18.57	0.023		
Congestive heart disease	8.15	0.007	9.86 (1.48 - 65.55)	0.018
RCRI				
≤ 2	1			
>2	8.50	0.049		
SAPS II	1.09	0.001		
APACHE II	1.51	0.001	1.40 (1.11 - 1.76)	0.005

^a ^Logistic regression analysis with stepwise forward method was used with an entry criterion of p < 0.05 and a removal criterion of p > 0.1.

SAPS, Simplified Acute Physiology Score; APACHE II, Acute Physiology and Chronic health Evaluation.

*Adjusted to age, renal insufficiency, congestive heart disease, RCRI, SAPS II and APACHE II.

### Functional capacity and ADL

Six months after discharge from the PACU, 62% and 20% of patients, respectively, were dependent in at least one activity in instrumental and personal ADL (Table 5).

**Table 5:** Dependency and self reported changes in health in general 6 months after PACU discharge (n = 50)

Variable	Before surgery (n = 50)	6 months after AFB (n = 50)	P
Personal activities of daily living			
**Katz scale**	0	0	**0.040**
Dependency in P-ADL, n (%)	1 (2)	10 (20)	0.796
Instrumental activities of daily living			
**Lawton scale**	**7.0 (7.0-7.0)**	**5.0 (3.8-7.0)**	**<0.001**
Dependency in I-ADL, n (%)	6 (12)	31 (62)	0.271

AFB, aorto-bifemoral bypass surgery; I-ADL, Instrumental Activities of Daily Living;

P-ADL, Personal Activities of Daily Living

Scores on the Katz and Lawton scales were significantly different after surgery, indicating more dependency. There were no differences in percentage of dependency for ADL-I and ADL-P.

#### Quality of Life Measures

Overall, 64% stated that their health in general was better on the day of testing and 8% considered it to be worse at that time than previously (six months before PACU discharge). There were no statistically significant differences between patients' baseline characteristics and worse self-reported general level of health.

Patients submitted to AFB because of occlusive disease had worse SF-36 scores for role physical (median 43.8 versus 75.0) and for general health perception (median 42.0 versus 50.0) than patients with aneurysmatic disease (Table 6).

**Table 6:** SF-36 6 months after AFB

Variable	Aneurysmatic disease	occlusive disease	p
SF-36 domains			
physical function	70.0 (40.0-75.0)	50.0 (20.0-70.0)	0.094^a^
**role physical**	**75.0 (37.5-81.3)**	**43.8 (18.8-75.0)**	**0.047^a^**
bodily pain	62.0 (51.0-84.0)	51.0 (41.0-74.0)	0.160^a^
**general health perception**	**50.0 (35.0-77.0)**	**42.0 (20.0-55.0)**	**0.048^a^**
Vitality	37.5 (20.8-58.3)	29.2 (20.8-41.7)	0.213^a^
social functioning	62.5 (37.5-87.5)	50.0 (37.5-75.0)	0.338^a^
role emotional	50.0 (33.3-100)	50.0 (25.0-75.0)	0.308^a^
mental health	52.0 (32.0-80.0)	48.0 (32.0-68.0)	0.378^a^

^a ^Mann-Whitney U test

SF-36, Short-form 36; AFB, aorto-bifemoral bypass surgery

Compared to values obtained from the general urban population of Porto, the SF-36 sub-scores of all patients submitted to AFB were worse in all domains (Table 7).

**Table 7:** SF-36 after AFB and population SF-36 means

Variable	After AFB (n = 50)	urban population (n = 1326)	p
SF-36 domains	mean ± sd	mean ± sd	
physical function	52.5 ± 27.5	75.4 ± 23.6	<0.001^a^
role physical	52.4 ± 32.9	76.7 ± 26.1	<0.001^a^
bodily pain	56.9 ± 26.1	65.7 ± 26.2	0.019^a^
general health perception	46.9 ± 25.1	59.5 ± 19.8	<0.001^a^
Vitality	34.8 ± 16.8	57.2 ± 21.1	<0.001^a^
social functioning	59.0 ± 26.5	76.0 ± 24.1	<0.001^a^
role emotional	54.0 ± 33.7	76.9 ± 25.8	<0.001^a^
mental health	49.7 ± 23.8	66.1 ± 22.8	<0.001^a^

^a^t test for independent groups

SF-36, Short-form 36; AFB, aorto-bifemoral bypass surgery

Patients submitted to AFB did not differ in any of the SF-36 domains from other PACU patients who were not submitted to AFB (Table 8).

**Table 8:** Results of SF-36 administration compared to results in surgical patients 6 months after PACU discharge

Variable	after AFB (n = 50)	after other PACU surgical patients (n = 737)	p
SF-36 domains			
physical function	55.0 (30.0-71.3)	55.0 (25.0-80.0)	0.811^a^
role physical	50.0 (29.7-81.3)	43.8 (18.8-75.0)	0.225^a^
bodily pain	52.0 (41.0-74.0)	52.0 (32.0-80.0)	0.984^a^
general health perception	45.0 (30.0-65.5)	45.0 (25.0-67.0)	0.863^a^
vitality	31.3 (20.8-42.7)	33.3 (20.8-45.8)	0.896^a^
social functioning	62.5 (37.587.5)	62.5 (37.5-87.5)	0.728^a^
role emotional	50.0 (25.0-83.3)	50.0 (25.0-83.3)	0.485^a^
mental health	48.0 (32.0-68.0)	52.0 (32.0-68.0)	0.854^a^

^a ^Mann-Whitney U test

SF-36, Short-form 36; AFB, aorto-bifemoral bypass surgery

## Discussion

AFB surgery is a current treatment for two types of complex disease: aorto-iliac occlusive and abdominal aortic aneurysms. This surgery is performed to improve long-term survival and to preserve function.

In the present study we found that independent predictors of mortality before six months after AFB surgery were higher APACHE II scores on admission to the PACU and congestive heart disease.

In a study published by Thomas S. Huber et al. [20], the authors studied aortic reconstructions performed for aneurismal or occlusive disease. They found that multiple systemic organ failure (MSOF) was a leading cause of death and admitted that patient age, APACHE II, and a diagnosis of sepsis at the time of ICU admission had previously been identified as risk factors for the development of MSOF. In that study the mortality rate did not vary with the indication for aortic reconstruction (aneurismal disease, 6.3%; occlusive disease, 5.7%; combined aneurismal/occlusive, 8.3%). In that study they also concluded that history of congestive heart disease was a predictor for mortality and the other predictors were patient age, low ejection fraction, duration of operative time, and performance of additional procedures.

Concerning only aortic occlusive disease, Morris-Stiff et al. [21] found a 30-day mortality of 10.4%, with a 1-year patient and graft survival of 80%; for open elective treatment of aneurismal disease but most large series describe hospital mortality rates of 3 to 6%[22,23] although the existence of pre-existing comorbidities were associated with higher mortality rates.

In the univariate analysis we identified six risk factors for mortality after AFB: Age, renal insufficiency, congestive heart disease, RCRI, SAPS II and APACHE II.

In the study of Dimick et al. [24] the authors also identified age of 65 years or older as a risk factor for mortality after AFB and Teufelsbauer et al. [25] found five independent predictors for mortality after open surgical repair of infrarenal aortic aneurysms. Two of these predictors were similarly found in our univariate analises: age and renal dysfunction. Beyond these they also found type of operation, pulmonary dysfunction and diabetes.

Our results differ from those of Wolters et al. [26], who studied ASA physical status, APACHE-II, and operative severity score for enumeration of mortality and morbidity (POSSUM) classification and SAPS, and concluded that none of the systems analyzed separately was useful for determining morbidity and mortality in patients after aorto-iliac surgery.

We evaluated health related quality of life six months after discharge from the PACU according to the American College of Surgeons and the American Society of Vascular Surgery, both of which have promoted the use of SF-36 in the surgical population [27,28]; this questionnaire has been validated for patients with vascular diseases [29].

In the present study we examined the effect of AFB surgery on quality of life and independence in activities of daily living. To study the impact of the procedure on quality of life we used the self-evaluated health transition item of the SF-36 questionnaire. This item is not used in scoring the scales but has been shown to be useful for estimating average change in health status over the year prior to its administration [30].

Although patients submitted to AFB because of occlusive disease were younger than those submitted to AFB because of aneurysmatic disease, they had lower SF-36 scores in two domains: role physical and general health perception. As role physical refers to the extent to which a respondent's performance of roles in daily activities is impeded by their physical state of health, and individual general health perception is measured by ratings such as excellent, very good, good, fair or poor, these differences may reflect the burden of dependency and the perception of a chronic disease.

Comparisons with a general (taken as "control") population are difficult to interpret because patients subjected to AFB generally perceive themselves as chronically ill. Thus, our finding that quality of life was worse in our patients than in the general population was not unexpected. A comparison with other PACU surgical patients with similar demographic characteristics from the same urban area seemed more appropriate for establishing comparisons with AFB patients, and the results, which showed no differences, are comprehensible.

The patients in our study had higher degrees of dependency in instrumental and personal ADL after surgery, which was not entirely unexpected in view of the extent of comorbidities and the natural history of their disease. It seems paradoxical that these patients, despite being more dependent, stated that their quality of life was better than before surgery. We think this could be explained by their expectation that surgical intervention would promote better health.

The limitations of this study include its retrospective nature, the relatively small number of patients and the fact that the patient population in the study is inhomogeneous. We have included patients submitted to ABF surgery done for aneurysmal aorto-iliac disease and patients with occlusive arterial disease. The first group of patients are asymptomatic and the procedure is prophylactic in its nature but in the second group ABF surgery is directed towards improvement of the arterial blood supply to the lower extremities, thereby improving the signs and symptoms. This may have influenced differences in the quality of life during the postoperative 6 month period between these two groups of patients.

Another limitation is that we did not apply the SF-36 questionnaire before surgery so it was not possible to compare quality of life of patients before and after surgery, as in the prospective study by Aljabri et al. [31] on patients after abdominal aortic aneurysm repair. Nevertheless, we used the SF-36 question about self-reported changes in health status ("compared to one year ago, how would you rate your health in general now?") to conclude that general health was better for most patients on the day they completed the SF-36 than before surgery.

## Conclusion

This study shows that congestive heart disease and APACHE II were considered risk factors for mortality at six months follow-up. Survivors who have undergone AFB perceive an improved quality of life although they are more dependent in ADL tasks and have worse scores in almost all SF-36 than the population to which they belong.

## Competing interests

The authors declare that they have no competing interests.

## Authors' contributions

All people listed as authors contributed to the preparation of the manuscript and no person or persons other than the authors listed have contributed significantly to its preparation.

Each listed author participated in the work to the extent that they could all publicly defend its content. They all read the manuscript before its submission for publication and are prepared to sign a statement stating they had read the manuscript and agree to its publication.

## Pre-publication history

The pre-publication history for this paper can be accessed here:

http://www.biomedcentral.com/1471-2261/10/15/prepub
